# Microglial regulation of synaptic plasticity in transsynaptic degeneration of glaucoma

**DOI:** 10.3389/fnins.2026.1828085

**Published:** 2026-05-28

**Authors:** Yingying He, Yu Dai, Yanxia Wu, Zhaorui Xu, Xuejing Lu

**Affiliations:** 1 Eye School of Chengdu University of TCM, Chengdu, China; 2 Eye Health with Traditional Chinese Medicine Key Laboratory of Sichuan Province, Chengdu, China; 3 Retinal Image Technology and Chronic Vascular Disease Prevention & Control and Collaborative Innovation Center, Chengdu, China

**Keywords:** Glaucoma, microglia, neurodegeneration, optic neuroprotection, synaptic plasticity

## Abstract

Glaucoma is a heterogeneous group of irreversible and blinding optic neuropathies caused by multiple factors. It is clinically characterized by progressive loss of visual field and decline in visual acuity, ultimately culminating in complete blindness. Hallmark pathological features include progressive degeneration of retinal ganglion cells and atrophy of the optic nerve. Importantly, the pathological process of glaucoma extends far beyond the eyeball, involving transsynaptic degeneration across the entire visual pathway. Microglia, as the principal immune regulators of the central nervous system, serve as the earliest sensors and effectors in the pathogenesis of glaucoma. By modulating synaptic plasticity, microglia contribute to synaptic loss and the disruption of neural circuits. They also play essential roles in maintaining neural tissue homeostasis. This review summarizes current evidence and underlying mechanisms of bidirectional transsynaptic degeneration in glaucoma. It highlights that targeting microglial functional homeostasis, particularly their regulation of synaptic plasticity, may be a promising strategy to mitigate glaucoma-associated transsynaptic degeneration and promote central neuroprotection.

## 1 Introduction

Glaucoma is a multifactorial neurodegenerative disease that has traditionally been regarded as an optic neuropathy predominantly driven by elevated intraocular pressure (IOP) and characterized by progressive degeneration of retinal ganglion cells (RGCs) (Jayaram et al., 2023). Accordingly, previous therapeutic strategies for glaucoma have largely focused on lowering IOP (Lusthaus and Goldberg, 2019). However, even when IOP is reduced to the normal range through pharmacological or surgical interventions, RGCs degeneration, apoptosis, progressive cell loss, and axonal loss often continue, and optic nerve damage as well as visual field deficits remain inadequately controlled (Garcia et al., 2019).

An increasing body of clinical evidence supports the notion that the neurodegenerative effects of glaucoma propagate extensively along the retina–thalamus–cortex visual pathway, exhibiting typical features of transsynaptic degeneration. This process involves anterograde degeneration spreading from the retina to higher-order visual centers, such as the lateral geniculate nucleus (LGN) and the primary visual cortex (V1), as well as retrograde degeneration that may be triggered by central injury or dysfunction, and in turn, adversely affect RGCs survival (You et al., 2021). These bidirectional cascading events suggest that the pathological mechanisms of glaucoma are far more complex than localized RGCs injury alone, involving multiple factors such as impaired axoplasmic transport, deprivation of neurotrophic support, and the propagation of pathological proteins. Transsynaptic degeneration may therefore represent a key mechanism underlying glaucoma pathology.

Microglia, as resident innate immune cells of the central nervous system, precisely regulate synaptic pruning and remodeling through their highly dynamic control of synaptic plasticity during development and disease, thereby ensuring the efficiency and fidelity of neural information transmission and playing a critical role in maintaining tissue homeostasis (Tremblay et al., 2010; Butovsky and Weiner, 2018; Andoh and Koyama, 2021). In the pathological environment of glaucoma, microglia become aberrantly activated, and their finely tuned synaptic regulatory programs may become dysregulated, thereby driving or exacerbating the cascade of transsynaptic degeneration (Au and Ma, 2022; Miao et al., 2023). In contrast, under pathological stimulation, activated microglia may also undergo adaptive functional phenotype transitions or exert neuroprotective and synapse-supportive effects by modulating a range of signaling pathways, including neurotrophic factors and the complement system. Through these mechanisms, microglia may help maintain the survival of a subset of RGCs and promote the stability of their synaptic connections during glaucoma progression.

This review aims to summarize the impact and underlying mechanisms of microglia-mediated synaptic plasticity in glaucoma-associated transsynaptic degeneration. Such mechanisms may represent potential therapeutic targets for the prevention and treatment of glaucoma and facilitate the development of optic neuroprotective strategies that extend beyond IOP lowering alone.

### 1.1 Methods and literature search strategy

This narrative review aims to summarize current advances in transsynaptic degeneration, microglia-mediated synaptic plasticity, and neuroprotective strategies in glaucoma. To enhance the transparency of literature selection, we conducted structured searches of PubMed/MEDLINE and Web of Science, supplemented by manual screening of reference lists from relevant articles. The search covered English-language publications from database inception to March 2026.

Search terms.

were constructed based on the core themes of this review and combined using the Boolean operators “AND” and “OR.” The search strategy included three main domains: (1) transsynaptic degeneration in glaucoma: “glaucoma” “ocular hypertension” “retinal ganglion cells” “optic nerve injury” “transsynaptic degeneration” “transneuronal degeneration” “anterograde degeneration” “retrograde degeneration” “visual pathway” “retina” “lateral geniculate nucleus” and “visual cortex”; (2) microglia and synaptic plasticity: “microglia” “synaptic plasticity” “synaptic pruning” “synaptic remodeling” “synaptic loss” and “complement”; and (3) neuroprotection and clinical translation in glaucoma: “glaucoma neuroprotection” “clinical trial” “drug delivery” and “translational medicine.”

Eligible studies included peer-reviewed English-language publications comprising clinical studies, histopathological investigations, animal and cellular experiments, review articles, and clinical trials. Priority was given to recent studies, while foundational studies relevant to key concepts were also retained. Studies were excluded if they were of low relevance to the topic, lacked clear mechanistic or clinical information, represented duplicate publications, or were published solely as conference abstracts, editorials, commentaries, or other non–peer-reviewed formats. Final study selection was based on thematic relevance, level of evidence, representativeness, and contribution to mechanistic understanding or clinical translation.

## 2 Transsynaptic degeneration in the visual pathway of glaucoma

See Figure 1.

**Figure 1:**
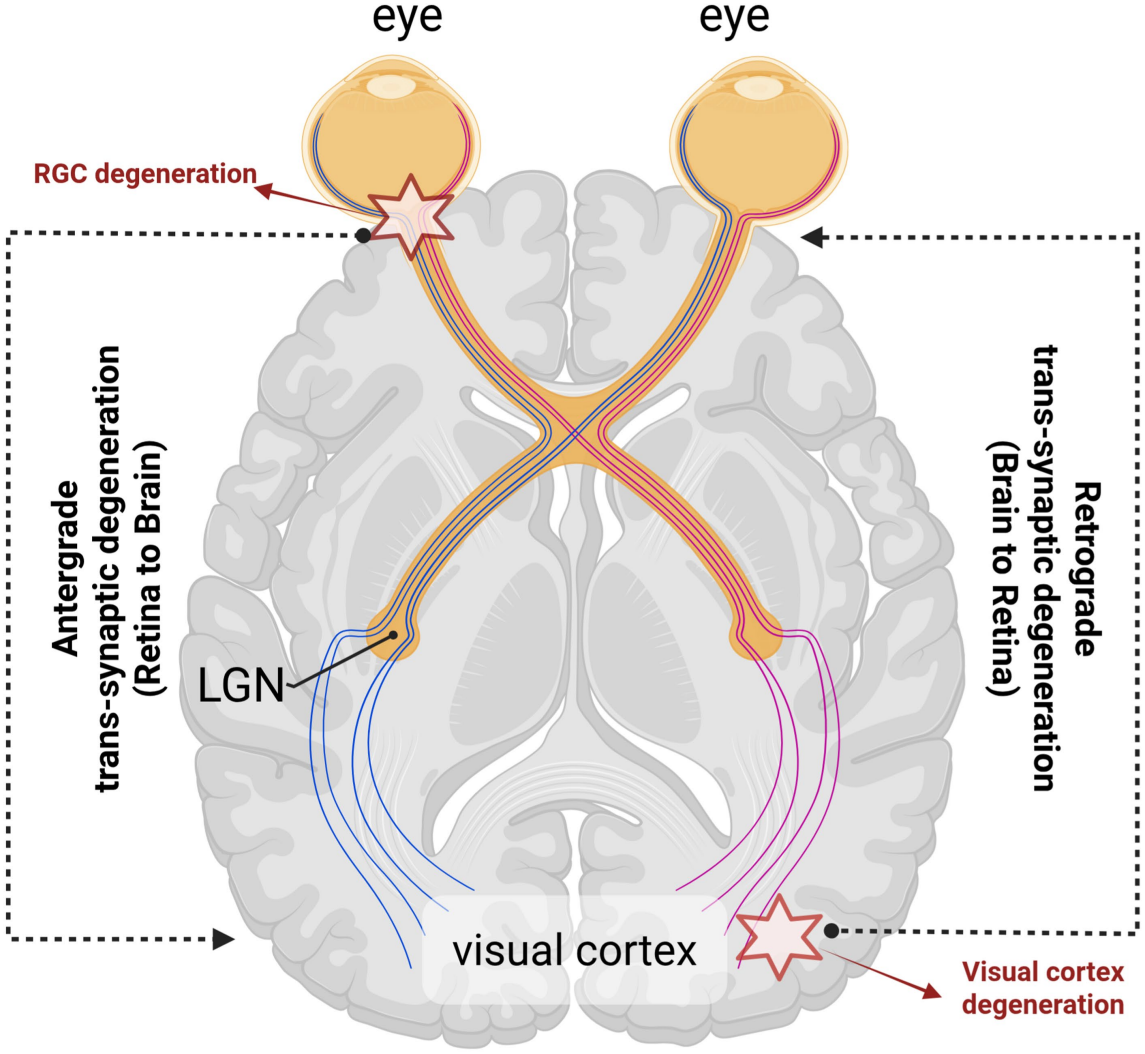
Transsynaptic degeneration in the visual pathway.

### 2.1 Transsynaptic degeneration in the visual pathway

Transsynaptic degeneration is a cascade-like degenerative process triggered by neural injury, in which damage propagates from the initial lesion site to anatomically and functionally connected regions within the central nervous system. As a result, neuronal network structures remote from the primary lesion progressively deteriorate. This process spreads bidirectionally through anterograde degeneration and retrograde degeneration. The underlying mechanisms involve multiple factors, including impaired axonal transport, propagation of pathological proteins, and disruption of the balance of trophic support (Fornito et al., 2015). Early evidence of such processes was reported by Nissl and colleagues, who observed chromatolysis in anterior horn cells within 24 h following peripheral nerve transection (Nissl, 1892), indicating an early morphological response of neurons to axonal injury. In 1963, Van Buren and colleagues demonstrated pronounced atrophy of the LGN and degeneration of the RGCs layer following occipital lobectomy and optic chiasm transection (Vanburen, 1963), thereby providing early evidence for retrograde transsynaptic degeneration within the visual system. These pioneering observations laid the foundation for understanding the bidirectional and cascading nature of transsynaptic degeneration. More recent studies have further demonstrated that degenerative changes can propagate bidirectionally along neuronal projections within the visual pathway (Balk et al., 2015; Hao et al., 2023).

The retina, as a functional component of the central nervous system, converts light signals into neural impulses and is physically connected to the brain via the optic nerve. The visual pathway originating from the retina can be anatomically divided into the optic nerve, optic chiasm, optic tract, LGN, optic radiation, and visual cortex (McCaa, 1982). The strong structural and functional connectivity between the retina and the visual cortex makes the visual pathway an ideal model for investigating the transsynaptic propagation of neurodegenerative processes. Within the visual pathway, injury to the retina or optic nerve can propagate anterogradely to involve the LGN, optic radiation, and V1, leading to neuronal atrophy, synaptic loss, and myelin abnormalities. Conversely, damage to the visual cortex can induce degeneration of the LGN and RGCs through retrograde mechanisms (Sharma et al., 2022). In glaucoma, multiple pathological factors disrupt axoplasmic transport and deprive RGCs of neurotrophic support, ultimately resulting in RGCs apoptosis. This degenerative process typically begins with axonal degeneration and loss of RGCs, subsequently affecting the optic nerve, LGN, and visual cortex (Di Ciò et al., 2020). Conversely, retinal degeneration has also been observed following injury to the V1 region of the brain (Yücel and Gupta, 2008), further supporting the bidirectional propagation characteristic of transsynaptic degeneration within the visual pathway.

In recent years, advances in neuroimaging technologies have substantially promoted investigations into structural and functional alterations along the visual pathway in patients with glaucoma. Clinical studies have employed optical coherence tomography (OCT) (Xie et al., 2022), advanced magnetic resonance imaging (MRI) techniques (Murphy et al., 2016), and single-voxel proton magnetic resonance spectroscopy to monitor degenerative changes between the eye and the brain and to detect structural or functional deficits (Bang et al., 2023; Luo et al., 2023; Zhu et al., 2025). Therefore, integrating multimodal imaging approaches to systematically evaluate alterations across the eye–brain axis is of great significance for improving our understanding of the mechanisms underlying transsynaptic degeneration in glaucoma, as well as for assessing disease progression and predicting prognosis.

### 2.2 Anterograde transsynaptic degeneration

Histopathological and clinical imaging studies in humans have provided important evidence of anterograde transsynaptic degeneration in glaucoma, characterising structural and functional alterations along the visual pathway. Early histological investigations demonstrated significant structural changes in the LGN of patients with glaucoma, including atrophy of tissue volume and layer-specific neuronal loss (Chaturvedi et al., 1993; Gupta et al., 2006). These findings offer direct morphological evidence; however, such studies are limited by the scarcity of donor tissue and small sample sizes. In addition, factors such as tissue procurement and ischemia may affect sample integrity, thereby constraining the extent to which these findings reflect *in vivo* pathological processes. Advances in neuroimaging techniques have provided noninvasive approaches to assess central nervous system alterations associated with glaucoma, thereby offering important clinical evidence. Zhang et al. (2015) reported a selective impairment of the magnocellular pathway in the LGN of patients with glaucoma, while no reduction in visual cortical responses was observed at early stages, suggesting stage-dependent differences in alterations across hierarchical levels of the visual system. Chen et al. (2025) further demonstrated LGN atrophy, decreased resting-state functional activity, and abnormal cortical functional connectivity in glaucoma patients. In addition, Duncan et al. identified changes in blood oxygenation level–dependent signals and cortical thinning in V1 in patients with primary open-angle glaucoma (Duncan et al., 2006; Yu et al., 2013). Collectively, these studies support an association between central neural alterations and retinal structural and functional damage in glaucoma. It should be noted that these imaging findings primarily reflect changes in neural activity rather than providing direct evidence of transsynaptic degeneration in the LGN or V1. Nevertheless, compared with anatomical approaches that directly assess neurodegeneration, neuroimaging offers significant advantages by enabling *in vivo* evaluation of functionally specific neuronal activity across multiple stages of the visual pathway in glaucoma.

Animal studies have provided biological evidence of anterograde transsynaptic degeneration in glaucoma by examining structures across different hierarchical levels of the central visual pathway. At the retinal level, glaucoma-like injury can involve multiple neuronal populations and induce early synaptic structural alterations. Zhou et al. (2019) reported that, in an acute ocular hypertension mouse model, neuronal apoptosis, axonal injury, and synaptic loss occur concurrently, affecting RGCs, amacrine cells, and cone photoreceptors. Further studies demonstrated that synaptic loss precedes dendritic retraction and RGC loss under elevated intraocular pressure, with more pronounced synaptic deficits observed in the OFF sublamina of the IPL (Ou et al., 2016). In the outer retina, photoreceptor loss can trigger structural synaptic plasticity in secondary bipolar cells, including dendritic remodeling and aberrant synaptic reconnection (Beier et al., 2018; Care et al., 2019). Della Santina et al. further focused on synaptic connections between specific RGC subtypes and bipolar cells, showing that, following transient intraocular pressure elevation, presynaptic ribbon loss at excitatory synapses of A ON-S RGCs precedes changes in postsynaptic density proteins. Concurrently, connections between A ON-S RGCs and T6 ON cone bipolar cells are reduced, whereas connections with rod bipolar cells are increased; however, this remodeling fails to restore light-evoked excitatory input (Della Santina et al., 2021). Subsequent studies examining the entire excitatory synaptic network within the IPL revealed sublamina-specific and asymmetric loss of both presynaptic and postsynaptic components, with presynaptic alterations occurring before or in parallel with postsynaptic changes (Soliño et al., 2023). These findings indicate that glaucoma-like injury induces early, selective, and temporally ordered synaptic disassembly and remodeling within retinal circuits, which may represent an early retinal circuit-level manifestation of transsynaptic degeneration in glaucoma. Chidlow et al. observed in an experimental ocular hypertension model that early RGC injury is characterized by rapid impairment of anterograde axonal transport and cytoskeletal damage at the optic nerve head. These changes occur prior to degeneration of distal axons and neuronal somata, exhibiting a spatiotemporal pattern that progresses from axons to cell bodies (Chidlow et al., 2011), thereby providing a potential basis for the propagation of injury to downstream neurons. Several studies have focused on alterations in the LGN in glaucoma. In experimental glaucoma models, changes in neuronal size, density, and number have been observed in the LGN, accompanied by degenerative damage in both the magnocellular and parvocellular layers (Weber et al., 2000; Yücel et al., 2001). These structural abnormalities are further associated with a range of molecular pathological changes, including glial proliferation and activation (Dai et al., 2011; Shimazawa et al., 2012), as well as oxidative stress–related DNA damage and repair responses (Yan et al., 2022), further supporting the notion that central neurons are directly affected and undergo injury in glaucoma. In addition, abnormal accumulation of Aβ and hyperphosphorylated tau has been identified in multiple visual centers in experimental glaucoma models, including the LGN, superior colliculus, and V1 (Chiasseu et al., 2016; Yan et al., 2017). These pathological changes have been implicated in RGCs apoptosis (Guo et al., 2007). Yan et al. further reported a progressive increase in the distribution of these abnormal proteins along the visual pathway (Yan et al., 2017), indicating a spatial gradient and suggesting their potential involvement in anterograde pathological spread. However, this evidence remains insufficient to demonstrate that such propagation is mediated via transsynaptic mechanisms. Studies of more distal visual cortex regions further show that, in experimental glaucoma models, V1 exhibits marked mitochondrial dysfunction, characterized by impaired complex II activity, reduced ATP production, and increased ROS (Hvozda Arana et al., 2021). These findings suggest that, from an energy metabolism perspective, central visual structures may be affected by upstream injury. However, these studies are insufficient to demonstrate that this process is mediated by transsynaptic mechanisms.

In summary, clinical and experimental studies collectively establish an evidence framework supporting anterograde transsynaptic degeneration from complementary perspectives: the former indicates the involvement of central visual structures, whereas the latter provides biological support for spatiotemporal progression and underlying molecular mechanisms. However, current evidence does not directly validate transsynaptic propagation, and its precise role in the pathogenesis and progression of glaucoma remains to be elucidated.

### 2.3 Retrograde transsynaptic degeneration

Current evidence for retrograde transsynaptic degeneration is largely derived from studies of central nervous system disorders. As an embryological extension of the central nervous system, the retina has been regarded as a unique “window” for monitoring intracerebral pathological processes (Grimaldi et al., 2018; Wang and Mao, 2021). Clinical studies using OCT have demonstrated that patients with post-geniculate visual pathway lesions, such as occipital lobe damage or stroke, exhibit secondary thinning of the retinal nerve fiber layer and the RGCs layer, which spatially corresponds to visual field defects (Molero-Senosiain et al., 2021; Maran et al., 2024). In neurodegenerative diseases such as Alzheimer’s disease and Parkinson’s disease, abnormal proteins including Aβ and tau not only accumulate in the brain but can also be detected in the retina (Bossy-Wetzel et al., 2004; Grimaldi et al., 2019; Indrieri et al., 2020). These findings suggest that central lesions may affect retinal neurons via retrograde pathways. Animal studies further support these observations at the structural level. Experimental injury at multiple sites along the visual pathway has been shown to induce chromatolysis in RGCs, accompanied by selective and age-dependent retrograde loss and degeneration (James, 1933; Leinfelder, 1938; Pearson et al., 1981; Tong et al., 1982). Consistent with clinical observations, lesions of the optic chiasm and occipital lobe in non-human primates result in severe RGC loss in the corresponding retinal regions (Vanburen, 1963). Collectively, these studies firmly establish the existence of retrograde transsynaptic degeneration in both animal and human visual systems. However, an important question arises: in glaucoma—a disease traditionally considered to be driven by elevated intraocular pressure and to progress from the retina toward the brain—does retrograde degeneration also contribute to its pathological process?

At present, direct clinical evidence demonstrating that central nervous system alterations in glaucoma can further aggravate RGCs injury through retrograde transsynaptic degeneration remains lacking. One clinical study reported that, even before the onset of visual field defects, patients with glaucoma exhibited inner retinal thinning, enlargement of the optic disc cup, and reduced visual cortical activity (Murphy et al., 2016). However, these findings do not directly demonstrate that abnormalities in the visual cortex can further exacerbate RGCs injury via retrograde transsynaptic degeneration. In comparison, the currently available supportive evidence is derived predominantly from animal studies. In the classic DBA/2 J mouse model of glaucoma, the pathological process does not begin in the retinal soma but is instead preceded by distal axonal degeneration. Studies have shown that optic nerve damage can be detected 1–2 months before measurable retinal injury (Schlamp et al., 2006). Axonal transport dysfunction and degeneration initially appear in the superior colliculus and progress in a distal-to-proximal manner (Crish et al., 2010). Other studies further support the notion that remodeling of RGCs dendritic circuits, axonal loss, and synaptic dysfunction occur before overt RGCs death in glaucoma 54. In addition, retrograde deficits at the optic nerve head have been observed to precede impairments in anterograde axonal transport (Martin et al., 2006). Some studies have also shown that distal axons are among the earliest structures to degenerate in chronic glaucoma (Sponsel et al., 2014). These findings provide indirect support for a retrograde injury mechanism from a temporal perspective. The long-term survival of RGCs is highly dependent on continuous neurotrophic signals supplied by their target neurons through retrograde axonal transport. Therefore, impairment of retrograde neurotrophic transport represents a key upstream mechanism leading to RGCs apoptosis in glaucoma. Multiple lines of evidence indicate that elevated intraocular pressure in glaucoma can obstruct the retrograde axonal transport of neurotrophic factors from the LGN to RGCs. The resulting deprivation of neurotrophic support subsequently triggers apoptosis and reduces the local capacity for neurotrophic factor processing, further disrupting anterograde transport along RGCs axons (Pease et al., 2000; Quigley et al., 2000). Overall, the evidence for retrograde transsynaptic degeneration in glaucoma is derived predominantly from inferences based on animal studies. It remains insufficient to directly demonstrate that central nervous system alterations can further exacerbate glaucoma progression through retrograde transsynaptic mechanisms, nor does it clarify whether central neurodegeneration can occur independently prior to RGCs loss.

## 3 Microglia

### 3.1 Physiological and pathological roles of microglia in the visual system

Microglia are the principal population of resident innate immune cells in the central nervous system and perform pleiotropic functions during neural development, homeostatic maintenance, and pathological responses. Through continuous surveillance of the microenvironment, microglia participate in maintaining neuronal network homeostasis (Cserép et al., 2019), support glial cell function (Du et al., 2022), and regulate critical processes such as synaptic pruning and remodeling (Paolicelli et al., 2011). These activities enable microglia to play key roles in maintaining tissue homeostasis and responding to injury within the visual system (Silverman and Wong, 2018; Reichenbach and Bringmann, 2019). Under physiological conditions, microglia typically exhibit a highly ramified morphology and continuously monitor the surrounding microenvironment through the dynamic extension and retraction of their processes. In pathological states, however, microglia display remarkable plasticity: they can rapidly become activated, alter their transcriptional and protein expression profiles, and ultimately transition into diverse reactive states that depend on the local microenvironment (Shemer et al., 2015).

During the pathogenesis of glaucoma, multiple stimuli—including elevated intraocular pressure, axonal injury, and metabolic imbalance—can trigger microglial activation, leading to morphological changes, increased secretion of inflammatory mediators, and alterations in phagocytic activity (Alarcon-Martinez et al., 2023; Miao et al., 2023). Activated microglia may promote neuronal apoptosis and axonal degeneration through the release of pro-inflammatory cytokines, reactive oxygen species, and complement proteins (Vecino et al., 2015; Au and Ma, 2022). In previous studies, microglia in glaucomatous injury have often been categorized as the pro-inflammatory M1 phenotype and the neuroprotective M2 phenotype (Zhang et al., 2023). M1 microglia secrete pro-inflammatory mediators, such as tumor necrosis factor-*α*, interleukin-1β, and inducible nitric oxide synthase, thereby amplifying inflammatory cascades. In contrast, M2 microglia express molecules such as CD206 and IL-10 and are involved in neuroprotection and tissue repair (Walker and Lue, 2015).

However, the traditional M1/M2 classification has limitations in explaining microglial functional states and cannot fully capture the complex, dynamic transsynaptic pathological processes involved in glaucoma, a chronic neurodegenerative disease. Recent advances in microglia research have proposed more exploratory perspectives on their phenotypic diversity. Single-cell technologies have revealed that microglia exhibit a dynamic, continuous spectrum of phenotypes across developmental, homeostatic, and pathological conditions (Prinz et al., 2019). For instance, rod-shaped microglia and disease-associated microglia have been identified and shown to be closely related to neurodegeneration (Wang et al., 2011). This study provides a systematic overview of the microglial phenotypic subpopulations currently reported and their characteristic features (Table 1). Although the specific roles of these newly defined microglial subpopulations in retinal development, homeostasis, and degenerative diseases remain under investigation, these findings highlight the remarkable heterogeneity and plasticity of microglial populations in response to diverse environmental signals.

**Table 1:** Overview of currently reported microglial phenotypes.

Microglial subtype/state	Key markers	Functional features	Evidence status in the visual system/glaucoma	References
Homeostatic microglia	P2RY12, TMEM119, CX3CR1	Immune surveillance; Tissue homeostasis; Synaptic monitoring; Neuro-support	Resident retinal microglia show homeostatic identity and niche-dependent specialization	Butovsky et al. (2013) and O'Koren et al. (2019)
Disease-associated microglia, DAM	TREM2, APOE, LPL, CST7	Downregulation of steady-state genes; upregulation of genes associated with lipid metabolism and phagocytosis; enhanced phagocytic activity.	TREM2–APOE-associated changes are reported in retinal degeneration and glaucoma, but full DAM staging remains incompletely validated	Keren-Shaul et al. (2017), Krasemann et al. (2017), and Margeta et al. (2022)
Neurodegenerative microglia, MGnD	APOE, CLEC7A, ITGAX, LGALS3	Overlaps partially with DAM; regulated by the TGF-β-APOE pathway; promotes neuroinflammation and neurodegeneration.	There are currently no direct reports regarding the retina or glaucoma.	Krasemann et al. (2017) and Xiao et al. (2024)
Rod microglia	primarily morphology-defined/Iba1, CD68	The cell bodies are elongated and rod-shaped, arranged along the neuronal cell bodies or axons; associated with neuronal damage and synaptic detachment	This has been observed in the dLGN of DBA/2 J mice and is associated with C1q/C3-mediated synaptic clearance	Taylor et al. (2014) and Thompson et al. (2025)
Proliferative-region-associated microglia, PAM	IGF1, SPP1, GPNMB, CLEC7A	Associated with the clearance of myelin fragments in developing white matter; exhibits strong phagocytic activity	As the optic nerve is a white matter structure of the central nervous system, PAM may play a role in its development and repair following injury; however, there is as yet no direct evidence to support this.	Hammond et al. (2019) and Li et al. (2019)
Pro-inflammatory/M1-like phenotype	iNOS, TNF-α, IL-1β, CD86, MHC-II	Release of pro-inflammatory mediators; Production of ROS; Promotion of neuroinflammation	Commonly reported in glaucoma and retinal injury models	Au and Ma (2022) and Li et al. (2024)
Anti-inflammatory/M2-like phenotype	CD206, ARG1, IL-10, TGF-β	Anti-inflammatory; Phagocytosis of apoptotic cells; Tissue repair; Neuroprotective support.	Commonly reported in glaucoma and retinal injury models	Au and Ma (2022) and Chen et al. (2022)

### 3.2 Microglia-mediated synaptic plasticity

**Figure 2:**
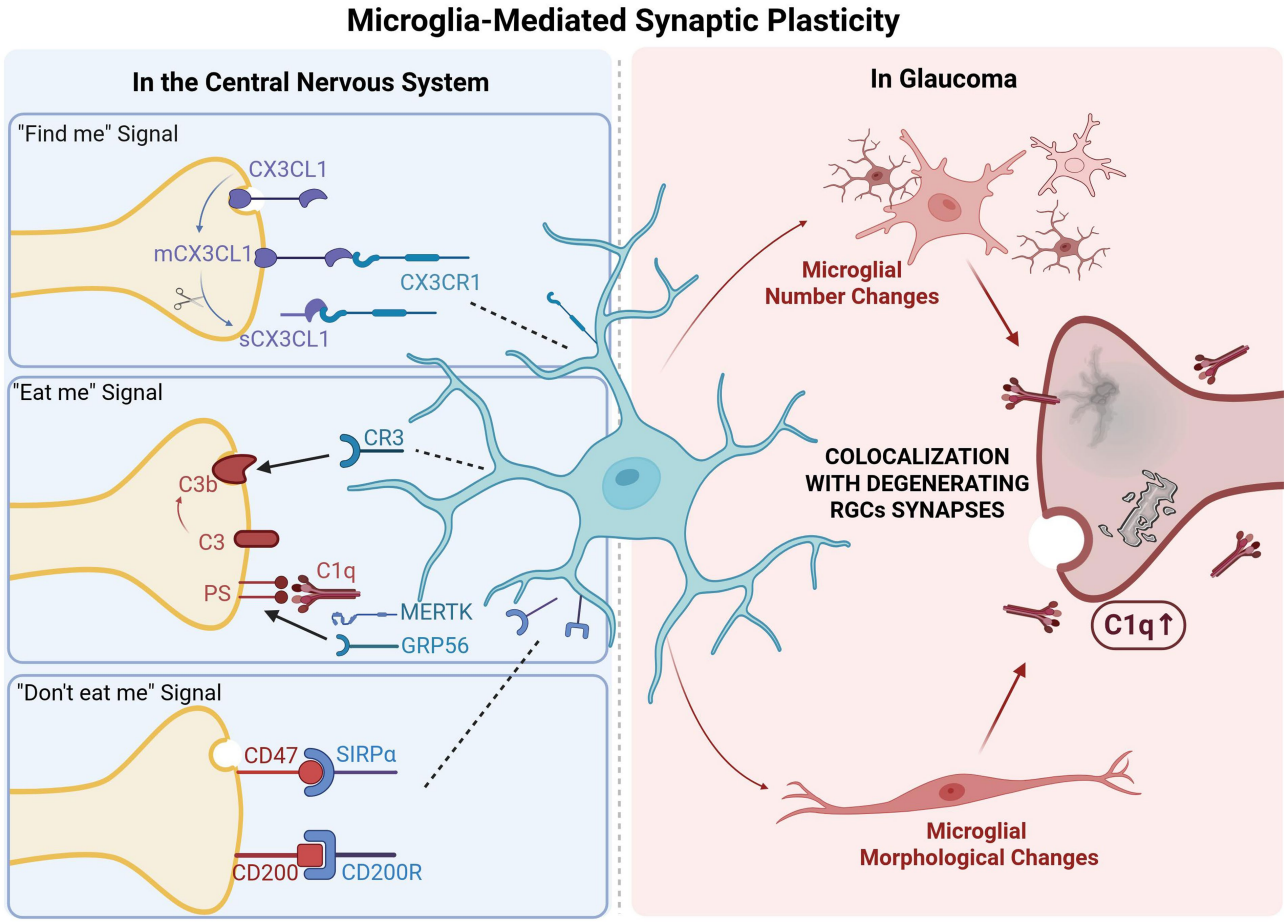
Microglia-mediated synaptic plasticity (schematic diagram, created with BioRender). Under physiological conditions in the CNS, microglia precisely regulate synaptic plasticity through multiple canonical signaling pathways. (1) “Find-me” signals: CX3CL1 exists in membrane-bound (mCX3CL1) and soluble forms (sCX3CL1), mediating adhesion and chemotaxis, respectively, both of which bind to microglial CX3CR1 to promote targeted recruitment to synaptic sites. (2) “Eat-me” signals: PS exposure on less active synapses is recognized by microglial receptors such as GPR56 and MERTK, promoting synaptic engulfment. Complement signaling also plays a central role, with C1q and C3 deposition facilitating C3b generation and binding to CR3 on microglia, thereby driving phagocytosis. (3) “Do not-eat-me” signals: neuronal CD47 and CD200 interact with microglial SIRP*α* and CD200R, respectively, suppressing excessive synaptic pruning. In glaucoma, microglia show increased numbers and morphological activation, colocalizing with degenerating RGC synaptic elements, alongside abnormal complement activation (notably upregulated C1q), suggesting their involvement in synaptic remodeling, although the precise mechanisms remain to be elucidated.

Synapses are the fundamental structures through which neurons transmit information. Dynamic changes in synaptic number, morphology, and transmission efficiency constitute the core basis of neural circuit plasticity. The ability of synapses to modify their function through structural remodeling and changes in transmission efficiency under specific conditions is called synaptic plasticity. Microglia are key regulators of synaptic plasticity, participating in the precise modulation of neural circuits by selectively eliminating excessive or functionally weak synapses through synaptic pruning (Zhou et al., 2018). Under pathological conditions, microglia-mediated synaptic pruning programs may become aberrantly activated, leading to the removal of both healthy and dysfunctional synapses, thereby impairing neural network function (Hong and Stevens, 2016; Stevens and Schafer, 2018) (see Figure 2).

#### 3.2.1 Microglia-mediated synaptic plasticity in glaucoma

In glaucoma, aberrant synaptic plasticity is considered one of the early key pathological events. Studies have shown that in DBA/2 J mice, loss of excitatory synapses and dendritic structural alterations occur prior to overt RGCs death (Berry et al., 2015), suggesting that synaptic abnormalities may contribute to the early stages of disease progression. Further ultrastructural studies provide preliminary evidence for the direct involvement of microglia in this process. Yu et al. observed an increase in microglial numbers following neuronal injury, along with colocalization of microglia with synaptic components on degenerating ganglion cell dendrites, predominantly at excitatory synaptic sites (Yu et al., 2025), indicating a potential role in the dismantling of synaptic connections. Thompson et al. (2025) reported that in the visual thalamus of DBA/2 J mice, microglia undergo a transformation toward a rod-like morphology and selectively eliminate RGCs input synapses through C1q/C3-mediated synaptic tagging and phagocytosis, leading to reduced synaptic connectivity and contributing to synaptic loss and circuit remodeling within the visual pathway. These findings are primarily based on structural colocalization and morphological observations of microglia–synapse interactions, suggesting that microglia regulate synaptic plasticity in glaucoma through morphological transformation and direct synaptic contact. However, the precise molecular mechanisms underlying microglia-mediated synaptic remodeling remain to be fully elucidated.

Previous studies have highlighted the important role of the complement system in this process, as evidenced by its expression, localization, and functional interventions. Stasi et al. (2006) reported an age-dependent upregulation of C1q expression in both human glaucomatous retinas and DBA/2 J mice, with localization to Müller cells and the inner limiting membrane. In contrast, Stevens et al. (2007) observed an early redistribution of C1q to synaptic regions of the retina in glaucoma, accompanied by significant synaptic loss and neuronal death. The discrepancies between these findings may be attributed to differences in disease stage and experimental models. Stasi et al. emphasized chronic changes associated with aging and disease progression, whereas Stevens et al. focused on the dynamic relocalization of C1q within synaptic regions during the early stages of the disease. In addition, variations in experimental models and detection methods may contribute to inconsistencies in the localization and expression patterns of C1q. Multiple studies have further supported the role of the complement system in glaucomatous synaptic injury, as evidenced by targeted interventions. Howell et al. (2011) demonstrated that genetic deletion of C1qa reduced the incidence of glaucoma and alleviated retinal synaptic damage, while Williams et al. (2016) reported that inhibition of C1 function, either genetically or pharmacologically, preserved dendritic and synaptic structures. These findings suggest that targeting the complement system confers neuroprotective potential in animal models; however, its efficacy and safety in clinical settings remain to be further established.

In summary, current research on microglia-mediated synaptic plasticity in glaucoma is derived predominantly from animal and cellular studies. Existing mechanistic insights are largely focused on microglial morphological and phenotypic changes, as well as the regulation of the complement system, while their roles at broader molecular levels remain to be further elucidated. Moreover, there remains a lack of direct clinical evidence, underscoring the need for further investigation and validation in clinical settings.

#### 3.2.2 Microglia-mediated synaptic plasticity in the central nervous system

Microglia-mediated synaptic plasticity has been extensively studied in the central nervous system. Given that glaucoma shares features with neurodegenerative diseases and involves the central visual pathway, these findings may provide a useful conceptual framework. However, these mechanisms should be interpreted with caution and not be considered as directly established in glaucoma. Their applicability to glaucoma remains to be further investigated.

The initiation of synaptic pruning depends on specific signals released by neurons to microglia. First, neurons recruit microglia to synaptic sites by releasing a variety of “find-me” signals. The chemokine fractalkine CX3CL1 is a transmembrane glycoprotein that can exist either as a membrane-bound form (mCX3CL1), mediating cell adhesion, or as a soluble form (sCX3CL1) generated by proteolytic cleavage, which exerts chemotactic activity (Winter et al., 2020). Both forms can bind to the specific receptor CX3CR1, which is expressed on microglia, thereby promoting microglial enrichment around synaptic structures (Paolicelli et al., 2011). In addition, different neurotransmitter signals, such as glutamate and dopamine, may confer selectivity to microglia during synaptic pruning (Haroon et al., 2016; Kopec et al., 2018). Further evidence from the study by Favuzzi et al. (2021) demonstrated that microglia can selectively eliminate inhibitory synapses through GABA-dependent signaling, highlighting the molecular specificity that governs microglia-mediated synaptic remodeling.

After localization to synaptic sites, microglia initiate the phagocytic program for redundant or low-activity synapses by recognizing specific “eat-me” signals. One important signal is the exposure of phosphatidylserine (PS) on the surface of low-activity synapses. This exposed PS can be recognized by specific receptors on microglia, such as GPR56 and MERTK, thereby mediating the selective elimination of excitatory or inhibitory synapses (Li et al., 2020; Park et al., 2021). The complement system provides another central regulatory pathway. During the development of the visual system, microglia mediate complement-dependent synaptic pruning to refine connections between RGCs and the LGN, thereby contributing to the establishment of single-RGC input (Stevens et al., 2007). Complement proteins C1q and C3 localize to synaptic terminals destined for elimination and serve as “eat-me” signals for microglia expressing complement receptor C3R (Schafer et al., 2012). C1q interacts with phosphatidylserine (PS), promoting the recruitment of C3 to synaptic sites, where it is subsequently converted to C3b. C3b then binds to complement receptor 3 (CR3) on the microglial surface, mediating synaptic engulfment.

Microglia-mediated synaptic pruning is also negatively regulated by “don’t eat me” signals, which serve to protect necessary synapses from excessive elimination and ensure appropriate synaptic competition during neural circuit formation. CD47 is a typical immune checkpoint molecule expressed by neurons, while its receptor SIRPα is expressed on microglia. Lehrman found that during development, CD47 expression in RGCs is upregulated, whereas SIRPα expression increases in a microglia-specific manner. Loss of CD47–SIRPα signaling enhances the synaptic phagocytic activity of microglia, leading to a reduction in functional synapses (Lehrman et al., 2018). Conversely, overexpression of CD47 suppresses microglial phagocytic function (Jiang et al., 2022). In addition, the interaction between neuronally expressed CD200 and microglia-expressed CD200R serves as a “do not eat me” signal, contributing to the protection of active synapses from phagocytic elimination (Lyons et al., 2017). In summary, these signaling axes function to limit excessive synaptic pruning and maintain synaptic homeostasis during neural circuit formation and in neurodegenerative processes.

Microglia can also regulate synaptic plasticity through multiple noncanonical mechanisms. Sipe et al. (2016) demonstrated that microglia participate in activity-dependent synaptic plasticity via the P2Y12 receptor, suggesting a role for purinergic signaling in this process, although the precise molecular mechanisms remain unclear. In addition, microglia can indirectly influence synaptic stability and plasticity by modulating the perisynaptic extracellular matrix and the neuronal microenvironment (Crapser et al., 2021). Microglia-derived neurotrophic factors further promote learning-dependent synapse formation and regulate synaptic plasticity and neural circuit function by activating neuronal TrkB signaling pathways (Parkhurst et al., 2013).

## 4 Neuroprotective strategies in glaucoma: from microglia-mediated synaptic plasticity to clinical translation

### 4.1 Microglia-mediated synaptic plasticity: preclinical studies

Microglia precisely regulate synaptic plasticity through diverse signaling mechanisms. However, research in glaucoma remains largely confined to the preclinical stage. Current evidence suggests that modulation of microglial phagocytic activity, phenotypic states, and secretory profiles may influence synaptic pruning processes, thereby intervening to some extent in the progression of RGC injury. Among these, neurotrophic factor signaling and the complement system represent two major mechanistic pathways that have been most extensively investigated, and may provide potential targets for preserving neural circuit integrity and slowing glaucoma progression.

Targeting the complement system represents one of the most extensively investigated intervention strategies in glaucoma research. Multiple studies have demonstrated that genetic or pharmacological inhibition of complement components can reduce synaptic and RGCs loss in experimental models of glaucoma. Williams et al. (2016) reported that C1q plays a regulatory role in RGCs synapse loss. Inhibition of the C1 complex, the initiating complex of the classical complement pathway, has been shown to prevent early synaptic and dendritic degeneration in glaucoma (Williams et al., 2016). Howell et al. (2011) reported consistent findings demonstrating that, in DBA/2 J mice, genetic deletion of C1qa or modulation of C1q expression in microglia at the ONH reduced the incidence of glaucoma and significantly attenuated optic nerve damage. Targeting downstream complement components may also mitigate neuronal loss. C3, a downstream molecule of C1q, has been reported to exert protective effects during the early stages of glaucoma, whereas its genetic deletion exacerbates optic nerve damage (Harder et al., 2017). Intravitreal administration of AAV2.CR2-Crry suppresses C3d deposition on RGCs and reduces both the rate and severity of RGCs degeneration (Bosco et al., 2018). Two studies investigating interventions targeting C3 have reported opposing outcomes, which may be attributable to differences in intervention strategies and disease stages. These findings suggest that the role of complement C3 in glaucoma may be both stage- and strategy-dependent: global inhibition may disrupt early physiological protective functions, whereas selective modulation of downstream pathological activation may be more favorable for neuroprotection. However, these conclusions require further validation. In addition, the C3a receptor, through interactions with IL-10 signaling and other immune-related pathways, has been identified as a major regulator of microglial reactivity and neuroinflammatory responses in glaucomatous mice (Harder et al., 2020). Intravitreal injection of anti-C5 antibodies has also been shown to prevent retinal degeneration and optic nerve injury in glaucomatous rats (Gassel et al., 2020), with its efficacy preliminarily validated in animal models.

Neurotrophic factor signaling is an important protective mechanism that regulates synaptic plasticity. Current studies are primarily focused on retinal development and the visual pathway, which may provide a reference framework for neuroprotective strategies in glaucoma. The neurotrophin family has been shown to regulate neuronal survival, development, and function through activation of tyrosine kinase receptors, playing critical roles in neuronal development, survival, and differentiation, as well as promoting synaptic plasticity and axonal regeneration (Weltman, 1987; Ma et al., 1998; Jeanneteau et al., 2020; Wang et al., 2021). Microglia are an important source of neurotrophic factors and profoundly influence the trophic support capacity of the local microenvironment. Neurotrophic factors bind to their specific tropomyosin receptor kinase (Trk) receptors and activate downstream signaling pathways such as MAPK/CREB, thereby regulating presynaptic neurotransmitter release, postsynaptic receptor function, and the synthesis and stabilization of synapse-associated proteins, ultimately maintaining synaptic efficacy and structural integrity (Hernández-Echeagaray, 2020). The application of hNGFp also plays an important role in retinal circuit maturation by activating microglia and the TrkA receptor. hNGFp selectively activates TrkA signaling and increases the expression of the axonal growth marker GAP-43 in injured axonal stumps, suggesting that NGF may exert neuroprotective effects on RGCs through microglial modulation (Latini et al., 2024). Studies have shown that exogenous BDNF supplementation upregulates the expression of synaptic vesicle proteins in RGCs and bipolar cells and increases the number of ribbon synapses in the inner nuclear layer. BDNF activates the downstream Akt signaling pathway and upregulates the expression of CaMKII and CREB in RGCs, leading to the accumulation of the downstream product F-actin around dendrites, suggesting that the CaMKII–CREB pathway participates in the regulation of dendritic spine structural and functional plasticity (Park et al., 2019). Although such exogenous interventions demonstrate the protective effects of neurotrophic factors on synapses, they do not clarify the mediating role of microglia in this process. In the LGN of DBA/2 J mice, microglia may mediate adaptive responses to elevated intraocular pressure by upregulating neurotrophin-3 and activating the downstream effector PLCγ1 (Thompson et al., 2025). Further studies are needed to perform functional validation to determine whether this adaptive response is specifically mediated by microglia. Together, these findings indicate that regulating the specific neurotrophic factor secretion profile of microglia is a key mechanism supporting synaptic functional remodeling. However, the current evidence is largely derived from single-factor interventions at single time points. The neuroprotective effects observed in animal models have not yet been adequately validated in clinical settings, and their long-term efficacy and safety remain to be established.

### 4.2 Clinical research and translational challenges in neuroprotection for Glaucoma

#### 4.2.1 Clinical research strategy and progress

**Figure 3:**
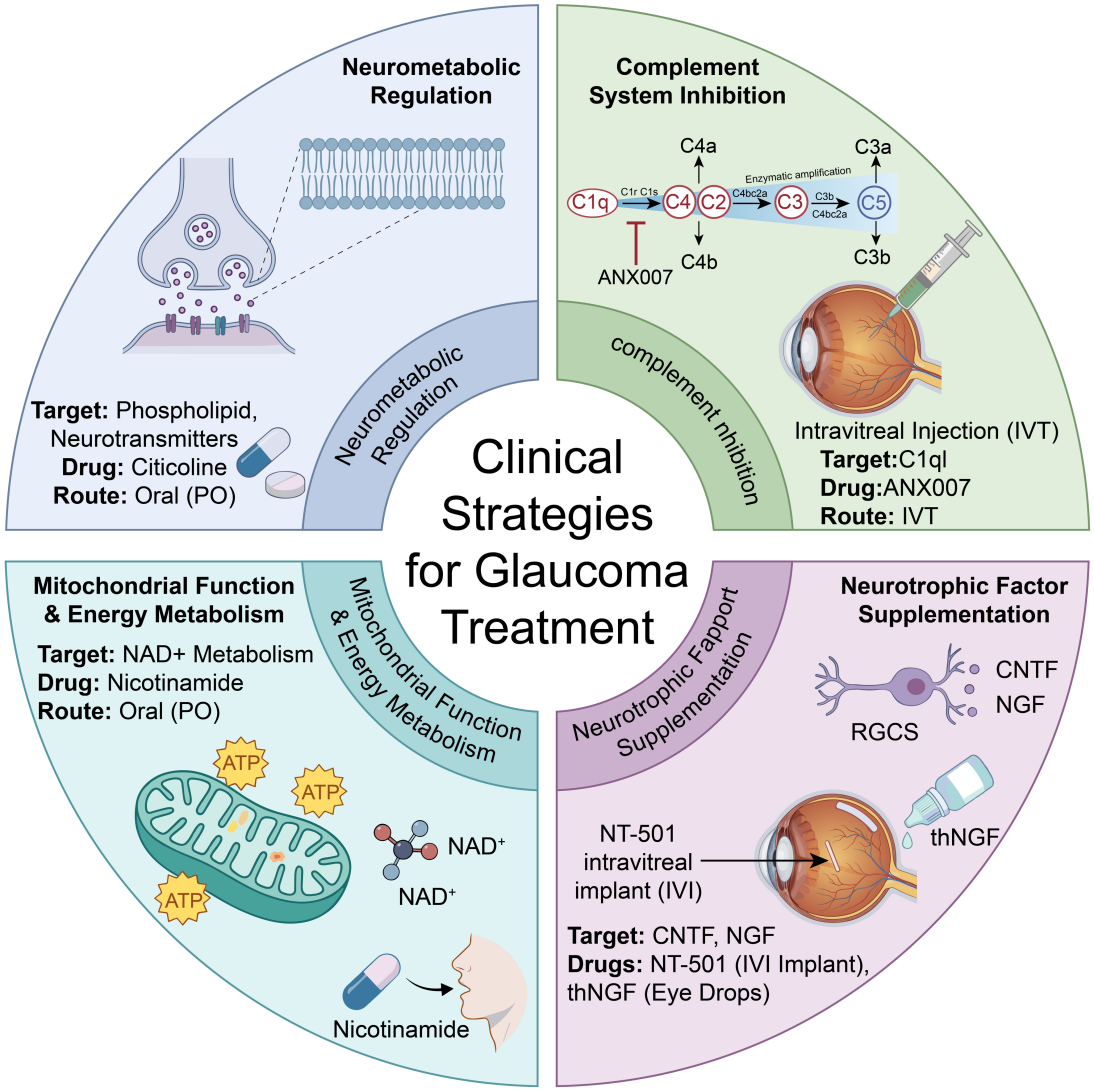
Recent therapeutic strategies for glaucoma. In recent years, clinical therapeutic strategies for glaucoma have included targeting the complement system, supplementing neurotrophic factors, modulating mitochondrial energy metabolism, and regulating neuronal metabolic pathways.

In recent years, clinical research on neuroprotection for glaucoma has become increasingly active, with multiple candidate therapies undergoing clinical trials employing diverse mechanisms of action and therapeutic strategies (Figure 3).

Regarding complement-targeted inhibition strategies, the C1q inhibitor ANX007 (Annexon Biosciences) has completed a phase I clinical trial. The results demonstrated that, across different injection frequencies and dosing regimens, treatment for 4 weeks significantly suppressed C1q activity (Sun et al., 2023), indicating favorable intraocular target engagement and pharmacodynamic effects. These findings are consistent with preclinical evidence suggesting that complement-mediated synaptic pruning contributes to the pathogenesis of glaucoma. However, the study did not demonstrate improvements in long-term visual functional outcomes, and its clinical efficacy remains to be validated in subsequent large-scale randomized controlled trials.

Neurotrophic and regenerative strategies are also being actively advanced. The ciliary neurotrophic factor (CNTF) sustained-release delivery system NT-501 achieves continuous intraocular release via intravitreal implantation, providing stable drug exposure while avoiding ocular surface toxicity. A phase I study has established its safety and reported partial structural and functional improvements, and it has now progressed to phase II clinical trials for further evaluation of efficacy (Goldberg et al., 2023). Noninvasive delivery approaches have also been explored. A phase Ib clinical study conducted by Beykin et al. (2022) showed that short-term, high-dose recombinant human nerve growth factor (rhNGF) eye drop treatment was associated with trends toward improvement in certain structural and functional parameters; however, due to the small sample size and nonrandomized design, these outcomes did not reach statistical significance, and the therapeutic efficacy remains uncertain. In addition, studies of topical insulin have suggested a reversible, short-term change in retinal nerve fiber layer thickness (Saludares et al., 2026), indicating potential neuroregenerative or neuroenhancing effects. Nevertheless, these findings are based on short-term observations, and evidence of long-term structural protection or effects on visual field progression remains lacking.

Targeting mitochondrial function and neuronal metabolism has emerged as another important direction in current research. Citicoline, a key metabolic intermediate involved in phospholipid synthesis and neurotransmitter regulation, has demonstrated multitarget regulatory potential in clinical studies. Anton et al. (2022) reported that oral citicoline combined with docosahexaenoic acid (DHA) improved the visual field index and its rate of change in patients with glaucoma. However, these findings were based primarily on short-term follow-up, limiting assessment of its impact on long-term disease progression. Furthermore, recent work by Parisi et al. (2026) showed that continuous oral administration of citicoline solution for 12 months not only improved RGCs function but also enhanced axonal conduction along the visual pathway, thereby ameliorating visual field defects. These results support the notion that citicoline may enhance retinal function through neuromodulatory mechanisms and influence neuroplasticity by altering post-retinal visual pathway connectivity. Studies on nicotinamide have focused on supporting RGC energy metabolism. A phase II randomized clinical trial conducted by De Moraes et al. (2022) demonstrated that oral nicotinamide combined with pyruvate improved certain visual function parameters in glaucoma patients in the short term, suggesting the potential for neuroprotection via enhancement of RGC energy metabolism. More recently, Shukla et al. (2025) further evaluated the safety, tolerability, and functional maintenance effects of this regimen over a longer follow-up period across different populations. In addition, nicotinamide monotherapy has also been investigated in normal-tension glaucoma (NTG), showing improvements in inner retinal function (Ha et al., 2026), supporting the concept that metabolic vulnerability represents an important and potentially modifiable component in glaucoma.

#### 4.2.2 Limitations of clinical research and challenges in translation

Although the neuroprotective strategies described above have shown some promise in early clinical studies, several prior trials have failed to achieve the expected therapeutic benefits, and substantial challenges remain in translating neuroprotection into clinical practice for glaucoma. The most representative failed example is the N-methyl-D-aspartate (NMDA) receptor antagonist memantine, which demonstrated inhibitory effects against glutamate excitotoxicity and protection of RGCs in multiple animal models of glaucoma (Hu et al., 2025). However, two large phase III randomized clinical trials failed to demonstrate a neuroprotective effect of memantine in slowing visual field loss in glaucoma (Weinreb et al., 2018). This failure may be attributable to the fact that targeting a single excitotoxic pathway is insufficient to address the multifactorial pathophysiology of glaucoma. In addition, because glaucoma progresses slowly and treatment effect sizes are often modest, conventional visual field endpoints may have limited sensitivity to detect neuroprotective effects, making genuine therapeutic benefits difficult to identify. Another controversial example is the α2-adrenergic receptor agonist brimonidine. The Low-pressure Glaucoma Treatment Study showed that, despite comparable intraocular pressure-lowering effects, the rate of visual field progression was markedly lower in the brimonidine group than in the timolol group (Krupin et al., 2011). However, the higher dropout rate in the brimonidine group due to allergic reactions may have introduced selection bias and thereby affected the reliability of the results. This suggests that methodological limitations may systematically overestimate treatment effects, and therefore the neuroprotective conclusions of this study should be interpreted with caution.

These findings indicate that substantial challenges remain in the clinical translation of neuroprotection for glaucoma. First, there are fundamental discrepancies between preclinical models and human disease. Rodents lack the lamina cribrosa structure characteristic of primates, which is considered a critical site for the initiation of axonal injury in human glaucoma (Burgoyne et al., 2005), thereby limiting the ability of such models to accurately recapitulate disease pathophysiology. In addition, most experimental models induce RGCs injury over relatively short time frames, making it difficult to simulate the chronic, slowly progressive, and multifactorial nature of human glaucoma, thereby restricting the clinical extrapolation of these findings. Second, the pathogenesis of glaucoma involves multiple interacting mechanisms, including mechanical stress, impaired blood perfusion, mitochondrial dysfunction, and inflammatory responses (Amato et al., 2021; Fernández-Albarral et al., 2024). Current neuroprotective strategies, however, are often directed at a single pathway, which may have limited efficacy in such a complex pathological context and may fail to produce stable, clinically meaningful therapeutic effects. This highlights the need to shift from single-target interventions toward multitarget or integrative regulatory approaches. Finally, regarding clinical endpoints and evaluation systems, most studies rely on visual field changes or retinal structural parameters as primary outcomes, focusing primarily on intraocular structure and function. Such measures may not adequately capture the integrated process of neural injury along the visual pathway from the retina to central visual structures. Although neuroimaging studies have begun to characterize visual pathway alterations in glaucoma, standardized acquisition and analysis methods are still lacking, and their correlation with functional impairment has not yet been validated in large-scale prospective studies. Therefore, these approaches cannot yet be considered reliable clinical endpoints for glaucoma.

#### 4.2.3 Clinical challenges: drug delivery and safety

In the clinical translation of neuroprotective strategies for glaucoma, drug delivery efficiency and long-term safety represent critical limiting factors. In particular, complement inhibitors and neurotrophic factor–based approaches, as discussed above, have demonstrated certain potential in preclinical and early clinical studies; however, their application continues to face significant challenges related to delivery and safety.

For complement inhibitors, as the therapeutic targets are located in posterior segment tissues such as the retina and optic nerve, intravitreal injection is currently the primary approach to achieve sufficient local drug concentrations. Although this route can enhance target engagement, the risks associated with repeated intraocular injections and intravitreal administration cannot be overlooked, including endophthalmitis, hemorrhage, and retinal injury (Patel et al., 2022). Moreover, the complement system plays a critical role in maintaining local immune homeostasis and host defense. Long-term inhibition may disrupt immune balance and increase the risk of infection or aberrant inflammatory responses (Liao et al., 2020). Therefore, while aiming to achieve neuroprotective effects, balancing therapeutic efficacy with immunological safety remains a key challenge for complement-targeted therapies.

Neurotrophic factors also face significant delivery barriers. Molecules such as CNTF and NGF are relatively large and exhibit limited stability, with poor penetration to the posterior segment via corneal or conjunctival routes, making it difficult to achieve sustained and effective retinal exposure through conventional topical administration. At present, delivery strategies primarily rely on intravitreal implants, encapsulated cell technology, and sustained-release systems to enable continuous drug release. Although these approaches can enhance local drug availability, their widespread application is limited by the surgical risks associated with invasive procedures and complications related to long-term implantation (Mallone et al., 2020). In addition, sustained or excessive expression of neurotrophic factors may induce aberrant glial activation and tissue remodeling (Do Rhee et al., 2022), highlighting the need for further optimization of dosage control and release kinetics.

In summary, drug delivery in glaucoma is constrained by ocular anatomical barriers, poor patient adherence, and treatment-related adverse effects (Lavik et al., 2011). Currently, a variety of novel delivery systems are under continuous development, including sustained-release implants such as Durysta and iDose TR, punctal plugs, and nanocarrier-based platforms (Goit, 2025), with the primary goals of prolonging local drug exposure, reducing dosing frequency, and improving patient compliance. In addition, the development and utilization of hybrid drugs and naturally derived compounds may enable multitarget synergistic effects in drug delivery (Bucolo et al., 2015; Pittalà et al., 2015; Godos et al., 2024). However, these strategies still face limitations related to invasiveness, long-term safety, and cost. Achieving efficient posterior segment–targeted delivery while minimizing treatment-associated risks remains a key challenge for future research.

## 5 Discussion

In recent years, glaucoma has increasingly been redefined as a central neurodegenerative disorder involving the entire visual pathway. At the structural level, multimodal neuroimaging has identified alterations in both structure and function across multiple levels of the visual pathway in patients with glaucoma. At the mechanistic level, the concept of transsynaptic degeneration provides a novel framework for explaining the progressive deterioration of visual function, marked interindividual variability, and continued disease progression observed in some patients despite adequate intraocular pressure control. Accordingly, the view of glaucoma as a neurodegenerative disease involving the central nervous system is becoming a widely accepted paradigm in the field. Within this context, microglia, as key regulators of synaptic homeostasis in the central nervous system, exhibit pronounced phenotypic and functional heterogeneity across different disease stages, anatomical locations, and microenvironments. Their roles in maintaining synaptic stability, remodeling neural circuits, and mediating neuroprotection are inherently dualistic. Targeting microglia-mediated synaptic plasticity therefore represents a promising new direction for neuroprotective interventions in glaucoma.

Although transsynaptic degeneration has emerged as an important theoretical framework for explaining glaucoma progression, its causal relationships and temporal dynamics remain controversial and incompletely understood. Substantial evidence has accumulated in support of anterograde transsynaptic degeneration in glaucoma. Both clinical imaging studies and experimental models have demonstrated that glaucoma-related neural injury can propagate along the visual pathway toward the visual cortex, from structural alterations to molecular pathological changes, indicating that retinal damage may lead to secondary involvement of central visual structures. In contrast, studies on retrograde transsynaptic degeneration in glaucoma remain limited. Direct clinical evidence demonstrating that central nervous system injury can drive RGCs loss in a retrograde manner is still lacking. Existing studies are largely based on structural correlations and molecular pathological observations, providing only indirect support for retrograde injury. Moreover, the mechanisms by which transsynaptic degeneration may exert reverse effects on the retina remain unclear, and its relative contribution to glaucoma progression remains uncertain. Regarding temporal relationships, current findings are also inconsistent. The traditional view holds that injury to RGCs and their axons represents the initiating event in glaucoma (Soto et al., 2008), with early manifestations characterized by impaired fast anterograde axonal transport at the ONH, exhibiting a spatiotemporal pattern extending from proximal to distal regions (Chidlow et al., 2011). In contrast, other studies have reported that axonal transport dysfunction may originate in distal target regions, such as the superior colliculus, and progress retrogradely toward the ONH, with distal axon degeneration preceding RGCs soma loss (Crish et al., 2010; Crish et al., 2010). These conflicting results may be attributable to methodological differences, including variations in animal models, injury paradigms, observation time windows, and the sensitivity of tracing techniques, reflecting an incomplete understanding of the initiation site and propagation direction of neurodegeneration in glaucoma. Nevertheless, it is increasingly recognized that glaucoma should be regarded as a neurodegenerative disease involving the brain (Chan et al., 2021).

There remain substantial gaps in understanding the role and mechanisms of microglia-mediated synaptic plasticity in glaucoma. From a technical perspective, synaptic plasticity is inherently characterized by high spatiotemporal specificity, and there is currently a lack of methodologies capable of dynamically tracking microglia–synapse interactions *in vivo* with high resolution, thereby limiting direct validation of causal mechanisms. In addition, microglia exhibit marked dynamism and heterogeneity in glaucoma. Single-cell transcriptomic analyses have identified multiple functional subpopulations, including homeostatic, disease-associated, and rod-shaped microglia. However, the spatiotemporal distribution of these subtypes across different stages of glaucoma, their patterns of phenotypic transition, and their specific effects on synaptic fate remain insufficiently characterized. More importantly, the regional specificity of microglia-mediated synaptic regulation has been largely unexplored in glaucoma. It remains unclear whether microglia employ similar recognition and pruning mechanisms across different synaptic types along the visual pathway, as comparative studies are lacking. If such mechanisms differ across anatomical levels, targeted intervention strategies may require region-specific design, which is particularly critical for understanding the role of microglia in the cascade of transsynaptic degeneration.

Overall, integrating microglia-mediated synaptic plasticity into the broader framework of transsynaptic degeneration in glaucoma may facilitate a systems-level understanding of disease onset and progression, and provide new perspectives for developing neuroprotective strategies beyond intraocular pressure reduction. Future studies should emphasize a comprehensive evaluation of the visual pathway from the eye to the brain to clarify the temporal relationships and causal links between retinal and central injuries. Such efforts may establish a more robust theoretical foundation for early diagnosis and neuroprotective interventions, thereby more effectively preventing disease progression and vision loss. At the mechanistic level, future strategies should focus on precisely modulating microglial functional states rather than simply suppressing their activation, to limit pathological synaptic loss while preserving their homeostatic support functions. With advances in single-cell omics, spatial transcriptomics, and in vivo imaging technologies, it is anticipated that the dynamic changes and specific roles of distinct microglial subpopulations during glaucoma progression can be elucidated at higher spatiotemporal resolution, enabling the development of more refined, stage-specific intervention strategies. From the perspective of clinical translation, neuroprotective approaches should aim at coordinated regulation of multiple pathological pathways, in combination with efficient and safe drug delivery systems, as well as the establishment of long-term follow-up and multidimensional functional evaluation frameworks to systematically assess efficacy and safety. Collectively, research centered on microglia-mediated synaptic plasticity holds promise for advancing glaucoma management from localized intraocular pressure control toward comprehensive neuroprotection of the visual system, thereby offering new directions for clinical intervention.
